# The 2008 Recession and Biological Health: Psychological Well-Being and Social Disadvantage Modify Vulnerability

**DOI:** 10.1093/geroni/igab046.816

**Published:** 2021-12-17

**Authors:** Julie Kirsch

**Affiliations:** University of Wisconsin-Madison, University of Wisconsin-Madison, Wisconsin, United States

## Abstract

Racial minorities and educationally disadvantaged experienced more housing loss, unemployment, and financial strain during the 2007-2009 Great Recession. These hardships may heighten stress and amplify persistent and growing health inequities, which were further worsened by the recent COVID-19 pandemic. It is therefore essential to identify factors that contribute to individual differences in vulnerability so that more effective interventions can be implemented, especially in older adult populations who may face unique economic hardships tied to age discrimination. According to the reserve capacity model, higher levels of psychosocial resources, including psychological well-being, can protect against the negative health outcomes related to heightened stress exposure. This study tested the intersections between recession hardship, pre-existing vulnerability defined as racial and educational disadvantage, and psychological well-being as predictors of biological indicators of chronic allostatic load. Chronic allostatic load was assessed with cardiovascular reactivity and recovery to acute mental stress and systemic inflammation (basal indicators of C-reactive protein and interleukin 6). Biological data came from a national sample of adults known as the Midlife in the US Study (MIDUS; age = 25-75, N=863) that completed assessments after the recession. Multiple regression models revealed that more widespread recession hardship predicted greater biological dysregulation. Tests of three-way interactions revealed that the association between recession hardship and biological dysregulation was strongest among respondents with combined disadvantages of low educational status and low levels of psychological well-being. This study connected a major economic event to individual variation in health vulnerability and identified potential biological pathways to future disease outcomes.

